# Code generation system based on MDA and convolutional neural networks

**DOI:** 10.3389/frai.2025.1491958

**Published:** 2025-03-11

**Authors:** Gabriel Vargas-Monroy, Daissi-Bibiana Gonzalez-Roldan, Carlos Enrique Montenegro-Marín, Alejandro-Paolo Daza-Corredor, Daniel-David Leal-Lara

**Affiliations:** ^1^Facultad de Ingeniería, Universidad Distrital Francisco José de Caldas, Bogotá, Colombia; ^2^Ingeniería de Sistemas, Facultad de Ingeniería, Fundación Universitaria Los Libertadores, Bogotá, Colombia

**Keywords:** deep learning, MDA, computer vision, artificial vision, generative programming, clean architecture

## Abstract

**Introduction:**

The software industry has rapidly evolved with high performance. This is owing to the implementation of good programming practices and architectures that make it scalable and adaptable. Therefore, a strong incentive is required to develop the processes that initiate this project.

**Method:**

We aimed to provide a platform that streamlines the development process and connects planning, structuring, and development. Specifically, we developed a system that employs computer vision, deep learning, and MDA to generate source code from the diagrams describing the system and the respective study cases, thereby providing solutions to the proposed problems.

**Results and discussion:**

The results demonstrate the effectiveness of employing computer vision and deep learning techniques to process images and extract relevant information. The infrastructure is designed based on a modular approach employing Celery and Redis, enabling the system to manage asynchronous tasks efficiently. The implementation of image recognition, text analysis, and neural network construction yields promising outcomes in generating source code from diagrams. Despite some challenges related to hardware limitations during the training of the neural network, the system successfully interprets the diagrams and produces artifacts using the MDA approach. Plugins and DSLs enhance flexibility by supporting various programming languages and automating code deployment on platforms such as GitHub and Heroku.

## 1 Introduction

The software industry has advanced rapidly through tools, methods, and technologies that improve scalability, adaptability, and efficiency. Despite these advancements, a critical gap remains in seamlessly integrating the planning and structuring stages with the actual implementation process in software development. For instance, manual coding from Unified Modeling Language (UML) diagrams—a standardized modeling language for system design that uses graphical notations to represent system architecture—requires repeated interpretation and adjustments. These processes not only consume time but also introduce potential inconsistencies. This disconnect highlights the need for innovative approaches to streamline these stages and minimize repetitive tasks (Richards and Ford, 2020; Pelliccione et al., 2023).

Our study proposes a novel framework that leverages computer vision, deep learning, and Model-Driven Architecture (MDA) to bridge this gap. These technologies were selected for their complementary strengths: computer vision allows for precise interpretation of architectural diagrams, deep learning provides robust pattern recognition and data processing capabilities, and MDA provides a structured approach to aligning abstract models with executable solutions. Together, they uniquely address the challenge of automating the translation of design artifacts into functional code, ensuring both accuracy and scalability. The proposed approach aims to reduce development time and improve accuracy by automating the generation of source code from architectural diagrams. Unlike existing solutions, which focus primarily on individual components such as code generation or diagram recognition, our framework integrates these processes into a cohesive system. This integration not only automates the conversion of diagrams into code but also ensures alignment with business logic and functional requirements from the outset (Abdelmoez et al., 2004).

The development of this prototype was driven by the academic objective of bridging the gap between system diagrams and the corresponding generated code. Traditional methods often fail to effectively demonstrate how high-level architectural designs translate into executable code, leaving developers with a limited understanding of this critical relationship. By leveraging the prototype as an experimental platform, this research seeks to illustrate and validate the potential of model-driven architecture (MDA) integrated with neural networks to automate and streamline these transformations.

This study presents a system that employs advanced image recognition and neural network techniques to process architectural diagrams and extract relevant information. The system translates this information into artifacts using the MDA approach. By combining modular design principles, asynchronous task handling, and plugin support for multiple programming languages, our solution offers significant improvements over current tools in flexibility, scalability, and automation.

## 2 Problem statement

The planning and structuring of software are critical for ensuring high-quality applications in the software development process. Tools such as Architecture Description Languages (ADLs) provide a valuable means to visualize and abstract system components. However, a significant disconnect remains between these high-level abstractions and their concrete implementation. This gap often results in manual intervention, which increases the likelihood of error propagation and extends development timelines (Tucker, 2004).

Furthermore, the iterative nature of modern software projects often introduces changes to initial designs, appearing as shifts in requirements, feature adjustments, or refinements based on stakeholder feedback. Existing tools struggle to accommodate these changes effectively due to their rigid workflows and limited adaptability to dynamically evolving project needs. While ADLs and related methodologies provide a starting point, they fail to provide the dynamic adaptability required for evolving requirements, complicating their integration into Agile workflows (Richards and Ford, 2020). The manual translation of architectural specifications into executable code not only consumes significant time but also creates challenges in maintaining alignment between business logic and technical solutions.

The research focuses on addressing these challenges with an innovative system. This system automates the translation of architectural diagrams into functional code, providing a seamless and efficient solution. By leveraging computer vision, neural networks, and the MDA framework, the proposed approach facilitates a seamless transition from planning to implementation, minimizing human error and ensuring compliance with initial project requirements (Pelliccione et al., 2023).

Moreover, existing tools lack the capability to dynamically adapt to software projects’ evolving requirements. This research addresses these issues by proposing a system that unifies planning, structuring, and implementation through automated code generation. The approach not only simplifies the development lifecycle but also ensures that architectural designs are directly translated into functional code, reducing errors and enhancing consistency (Pelliccione et al., 2023).

## 3 Background

Modern technology has led to the development of some useful tools for process automation, code generation, and development verification, among other system utilities for developers and non-developers, that allow them to choose between a number of uses and strategies to reach a similar goal with the ability to form automated processes. Therefore, developing a clear stage and software for this purpose is important.

### 3.1 AI and artificial vision

Artificial intelligence is a field of study whose definition varies according to the approach taken, including *thinking humanly, thinking rationally, acting humanly, or acting rationally* (Russell and Norvig, 2015). This variation may lead to debates in the definition process, where such perspectives are not the only obstacles to establishing a definition; the processes by which intelligent behavior and results can be achieved may also pose challenges (Artificial Intelligence Applications and Innovations, 2022). Through various lenses, several fields contribute to the definitions of belief and behavior recognized in these domains, with machine learning playing a crucial role in facilitating essential processes within intelligence, including learning. The expression of intelligence links results to interactions with humans; therefore, these should be considered in fields such as n*atural language processing*. Representations are naturally understood by humans, allowing for the extraction of valuable information. Consequently, it was important to develop projects that initially had a direct impact on text generation. One such project is GPT-2, released with multiple variations in the amount of data used for training; the largest version contains 1.5 billion input data points (Radford et al., 2019), developed by OpenAI. This was followed by the release of GPT-3, which has 175 billion parameters, with its experimentation reflecting OpenAI’s preparedness due to its high performance in natural language processing across various tasks, such as translation, questionnaire completion, and text formulation (Brown et al., 2020). This advancement has led to the development of several projects, including natural language to code generation (Shankar et al., 2020). Parallel projects have emerged in the field of computer vision, such as” *GPT Image”* (OpenAI, 2020), which employs unsupervised analysis to generate coherent pixels, thus creating images (Chen et al., 2020). Similarly, it is essential to acknowledge projects focused on code generation related to user interface design through the creation of mockups that outline their structure, including “Sketch 2 Code” proposed by Microsoft AI Lab in 2019 (Microsoft, 2018), which utilizes artificial intelligence to transform handwritten drawings into HTML prototypes. Designers exchange ideas on a whiteboard, and any modifications are instantly reflected in the browser using optical character recognition (OCR). However, in the Azure Cloud platform, returned objects assist in identifying the design, which is then transmitted to the Sketch 2 Code web application for the corresponding HTML generation (Robinson, 2019), a model that is followed by multiple competitors, such as “Editor,” among others.

### 3.2 Case tools

Computer-aided software engineering (CASE) tools are a set of software tools that can increase in terms of time in each of the stages of the development life cycle, with the ability to generate code based on a diagram or model. Therefore, the ability to generate code, as well as a method to keep the system updated with the ability to grow from diagrams and always help analysts identify gaps in security or improvement opportunities that with other systems is difficult. The architects using such systems can allow the flow of the system, make decisions with each of the stakeholders, and solve problems of depth (Jabbari et al., 2021).

This type of tool is highly capable of integrating into Agile development, allowing the generation of audit reports easily without harming or delaying the development team. In addition, it allows the automation of processes that can become tedious during quality verification; however, it also allows a software architect to generate codes under certain constraints, implement them, and verify them through test cases (García-Holgado and García-Peñalvo, 2021).

### 3.3 No code

The NO CODE Movement, along with its growing philosophy, offers a range of tools for both developers and non-developers that allow them to create software solutions largely without coding. Therefore, it is important to bridge the technological gap that persists today regarding the development role’s capacity in creating software solutions for the public. This initiative will empower individuals who may not understand or prefer not to use programming languages to easily generate such solutions through simple graphical interfaces, allowing end users to achieve results more quickly than conventional methods (Cypher et al., 2010).

Currently, the *NO CODE movement*, as a trend, is one of the most developed tools available in the market for users who seek its benefits while recognizing its potential drawbacks. Organizations aiming to enhance their development capabilities are actively working on many of these tools, and additional ones are being created to establish clear guidelines for customers. A design prototype that can be developed in real-time, fostering better understanding between clients and developers, is crucial (Spiridonov, 2021).

## 4 Theoretical framework

### 4.1 Computer vision

Computer vision is the processing of data from any modality that uses the electromagnetic spectrum to produce an image (Artificial Intelligence and Robotics, 2022). It is essential to highlight artificial vision as a concept that refers to the processing of sensor data, enabling a machine to extract useful information to fulfill a specific function (Biosensors in Food Safety and Quality, 2022). While there are some differences between them, these terms are often used interchangeably. In computer vision, a machine (typically a computer) automatically processes an image and answers the question, “What’s in the image?” (Szeliski, 2022). Key elements in these processes relate to digital image processing, which involves implementing processes with images as both inputs and outputs. Although there is no universal theory or methodology applicable to digital image processing (Dougherty, 2020), a method is needed to interpret data derived from an image by employing various techniques closely associated with image processing, such as:

- Convolution.
- Thresholding method.
- Feature detectors.
- Ramer–Douglas–Peucker algorithm.

### 4.2 Neuronal networks

Neural networks are computational models inspired by the nervous systems of living beings, which have the ability to acquire and maintain knowledge based on the information from which they are constructed. They can be defined as a set of processing units represented by artificial neurons, interconnected through multiple connections (artificial synapses), and implemented using vectors and matrices of synaptic weights (Artificial Neural Networks in Pattern Recognition, 2020). Therefore, within the standard operation of different neural networks, regardless of the architecture implemented, the *Perceptron*, created by Frank Rosenblatt in 1957, exemplifies this concept using numerical inputs and outputs. Each input connection is associated with a weight (Géron, 2022).

Therefore, among the exposed values, it is important to acknowledge the step function, or Heaviside, as the activation function and weight as *Z*:


$$z=w_{1}x_{1}+w_{2}x_{2}+\ldots +w_{n}x_{n}=X^{T}\;W$$

Therefore, different neural networks are set with various layers and connection patterns that follow the same signal propagation through the network. Real numbers determine the strength of connectivity between the two connected neurons because each signal that is transmitted through the connection is multiplied by the weight associated with the connection. Considering that the weights can be positive or negative depending on the case is essential. Therefore, it is necessary to recognize the adjustment of these weights. This is essential for the proper functioning of the network, which can be achieved through the training phase in which the weights are adapted with the so-called *learning rule*. This mathematical logic method improves the performance and training time from the update of weights and bias levels on the network when simulated in an environment with specific data or training (Handbook of Chemometrics and Qualimetrics, 1998). Consequently, it becomes essential to consider the definition of a new concept that will include a deeper area of study with respect to neural networks and deep learning, which is a learning approach using multilayer artificial neural networks that are trained by establishing weights and trade-offs in each layer by optimizing a loss function (the delta between the actual result and the predicted result). Thus, the neural networks of a deep learning model have a series of layers, starting with the input layer, followed by several hidden layers, and culminating with an output layer. It updates the network weights through different iterations to minimize the loss function that defines the aggregate difference between the model’s predictions and the actual values of the results in the training data set (Ryan, 2020). Based on the diversity of architectures in the process of construction and definition of the model, the following neural networks have been developed:

1. *Convolutional Neural Networks:* Convolutional neural networks are a type of neural network used for data processing characterized by a grid or two-dimensional topology. Notably, their name indicates the inclusion of mathematical convolution in at least one layer’s processes, as opposed to matrix multiplication (Aggarwal, 2023). It is essential to emphasize their superior performance in analyzing gridded data compared to other methods. Within the structure of these grids, it is possible to classify various types of layers, which are as follows:

- Convolutional Layer: this is the layer that defines a CNN as such and is where most of the computations occur. It requires different input data, a filter or kernel, and a feature map. The convolution process is achieved by implementing the filter on the receptive fields of an image, thus checking if a certain feature is in the image. It is essential to recognize the feature detector as a two-dimensional matrix of weights representing part of the image; thus, the kernel is applied to an area of the image, and a dot product is calculated between input pixels and said kernel, producing an output matrix. When repeating the process over the entire image, a set of dot products of the input and the kernel are generated, which is known as the *feature map, activation map, or convolved feature*. Thus, having implemented the convolution process on the image, a rectified linear unit transform (ReLU) is applied to the feature map, thereby introducing non-linearity into the model. However, within the parameters used within the convolution, there are several filters and, as previously mentioned, even different layers where the procedure is implemented, which, according to the processing of the procedure, eventually create a hierarchy of features within the CNN itself.
- Pooling layers: These perform a dimensionality reduction by reducing the number of parameters in the input, which, similarly to the convolutional layer, implements a filter through the entire input. However, this filter, previously called a kernel, does not contain weights; instead, the kernel or filter applies a grouping function to the values within the receptive field, populating the output matrix. It is necessary to mention the different types of grouping, such as:
  1. Average pooling: when the filter is scrolled through the input, it selects the pixel with the average value within the receptive field to send to the output matrix.
  2. Max pooling: as the filter scrolls through the input, it selects the pixel with the maximum value to send to the output matrix. Being more used than the *average pooling*.

Therefore, although more information is lost in this layer, it is fundamental in reducing complexity, improving efficiency, and limiting the risk of overfitting.

- A fully connected (FC) layer, as its name says, is based on the connection between the different nodes in each layer. It performs the classification task based on the features extracted from the previous layers with their respective filters; in contrast to the convolution and pooling layers that usually implement ReLu functions, these usually utilize a softmax activation function to classify the entries appropriately, producing a probability between 0 and 1 (IBM, 2020).

However, when defining a model to be trained, it is essential to acknowledge techniques and methodologies of deep learning, such as:

1. *Transfer Learning:* Transfer learning is a methodology where a model trained for one task serves as a starting point for a model performing another similar task. This approach is easier and faster because it allows for updating and retraining a network with transfer learning rather than training it from scratch. It is often utilized in object detection, image recognition, and speech recognition applications, leveraging models developed by the deep learning research community, such as widely used architectures such as GoogleNet and ResNet (MatLab, 2022a). The new structured networks include:

- ResNet50: This is a 50-layer deep convolutional neural network. You can load a pre-trained version of the network that has been trained with over a million images from the ImageNet database (MatLab, 2022b). ResNet, which stands for Residual Networks, is a classical neural network used as a backbone for various computer vision tasks. This model won the ImageNet challenge in 2015. The fundamental breakthrough of ResNet was its ability to successfully train extremely deep neural networks with more than 150 layers. Before ResNet, training deep neural networks was challenging due to vanishing gradients (Dwinvedi, 2022). Therefore, it incorporates numerous layers stacked and trained for specific tasks; thus, the network learns multiple low, middle, and high-level features by the end of its layers. In residual learning, instead of solely learning features, it focuses on learning residuals. A residual can be understood as the subtraction of the learned feature from the input of that layer. ResNet employs this method using direct access connections, which directly connect the input of layer n to layer (*n* + *x*).

Training this type of network has been shown to be easier than training simple deep neural networks and effectively addresses the issue of accuracy degradation (O. Foundation, 2022).

### 4.3 Natural language processing

Natural language processing is the processing of human communications by machines. It aims to teach machines to process and understand the language of humans, thereby enabling a communication channel between humans and machines. The key to the above definition of natural language processing is that communication has to occur in the natural language of humans, thereby enabling an efficient communication channel between the two. However, natural language processing is necessary, and machines, machine learning, and deep learning models especially work best with numerical data. Numerical data is difficult for humans to produce naturally; thus, natural language processing works with textual data and converts it into numerical data, allowing machine learning and deep learning models to fit it. It bridges the communication gap between humans and machines by considering the spoken and written forms of human language and converting them into data that machines can understand (Raaijmakers, 2022).

### 4.4 Model driven architecture (MDA)

The framework known as model-driven architecture consists of practices at the software architecture level, focusing on designing a layer above the business layer or domain entities. Its objective is to ensure that the software solution is not tied to a specific technology, allowing for independent decisions regarding this aspect.

The initiative produced by the Object Management Group (OMG) to increase productivity and reuse is based on the separation of abstraction and responsibility within the development scope. Its main objective is to isolate the domain-specific behavior of any problem in an abstraction extension to the generic solution that should be supported by a preliminary design.

**Figure 1:**
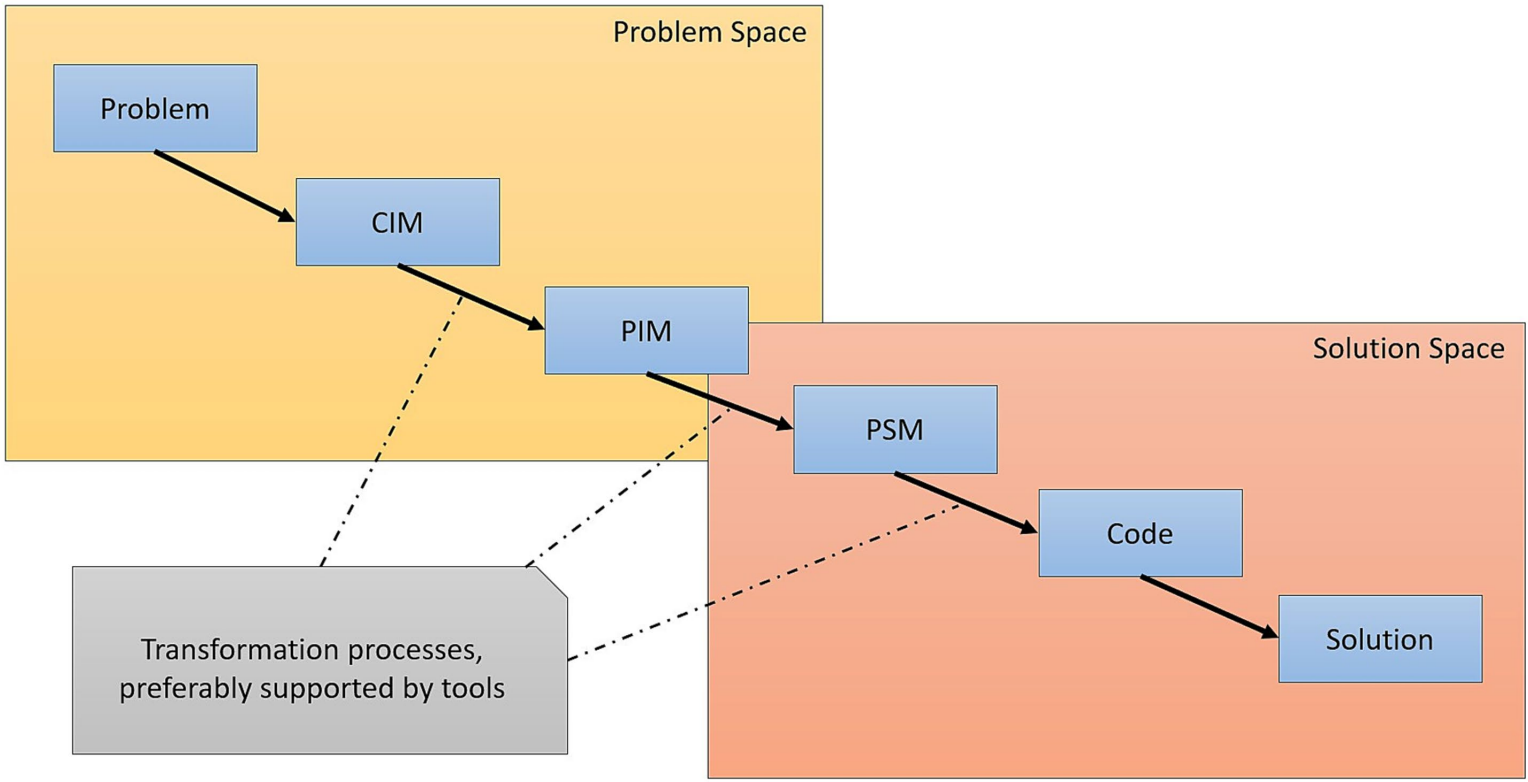
Layers of an MDA solution (Moreno-Rodríguez, 2020).

Therefore, MDA is an open, vendor-neutral architectural framework that leverages all associated OMG standards (Figure 1). MDA supports the application domains being analyzed through this process (Moreno-Rodríguez, 2020).

Based on the stages of MDA development involving the process and analysis of the following models:

- CMI Computational Independent Model. This model focuses on system requirements and is the basis of the core business.
- PMI Platform Independent Model. It represents the business process model.
- PSM Platform Specific Model generates multiple PSMs and targets a specific domain language.

### 4.5 Event-driven architecture (EDA)

As more software applications need to scale horizontally rather than relying on a monolith that allows only vertical growth, some of their modules were adapted independently, enabling them to develop horizontally per module. This approach generated the previously mentioned service-oriented architecture (SOA). While this provided an excellent architectural tool that can expand sufficiently at the domain module level, communication between services became more complex. This indicated that its own architecture was necessary for communication between services A and B. Consequently, the event-driven architecture (EDA) is introduced.

**Figure 2:**
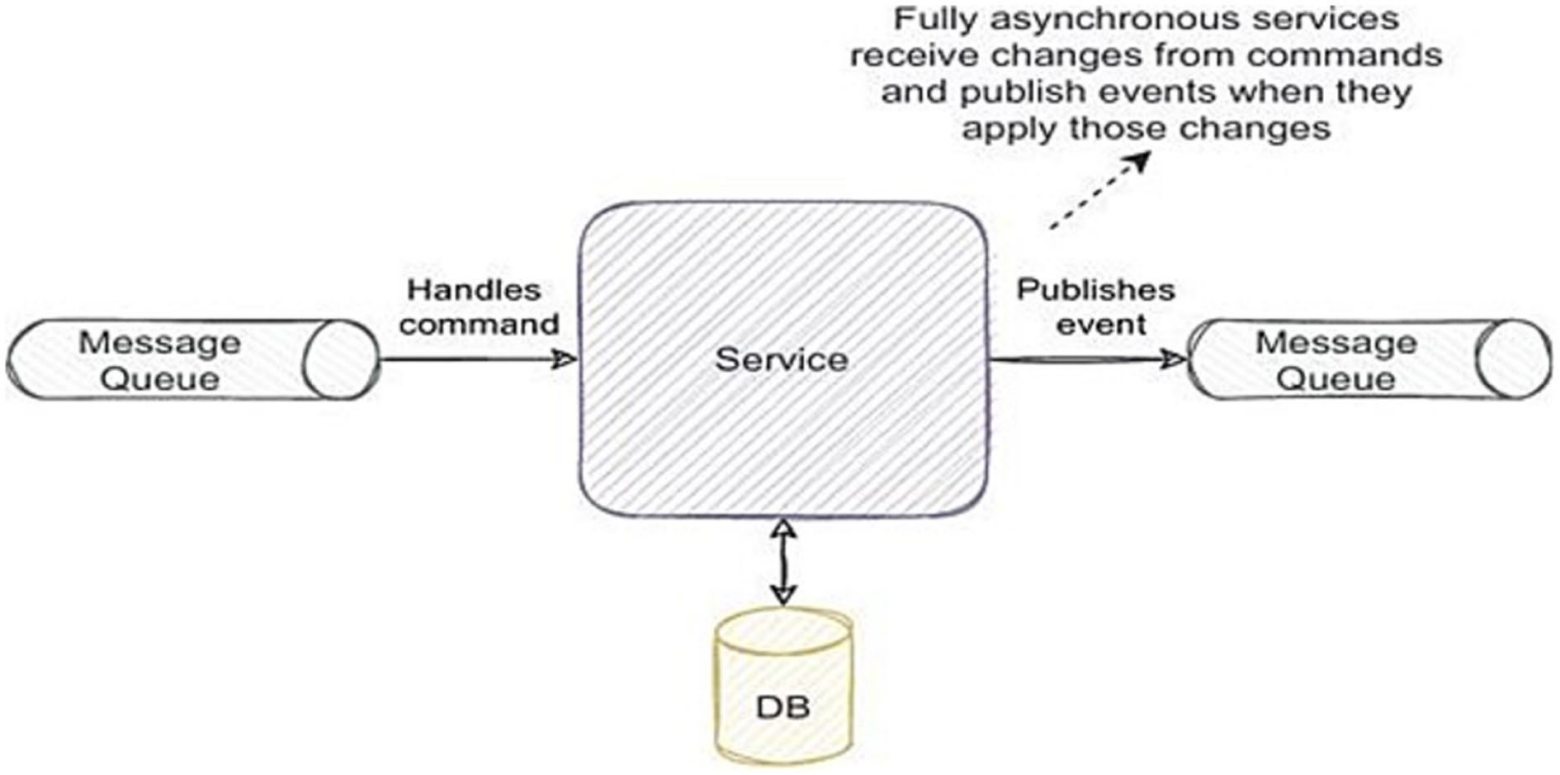
Asynchronous PUB/SUB structure of a service in EDA (Rocha, 2021).

The solution requires a messaging bus layer that operates asynchronously, utilizing a Publisher/Subscriber pattern or streaming solutions. This setup facilitates communication between each of the organization’s services and even third-party services (Figure 2).

**Figure 3:**
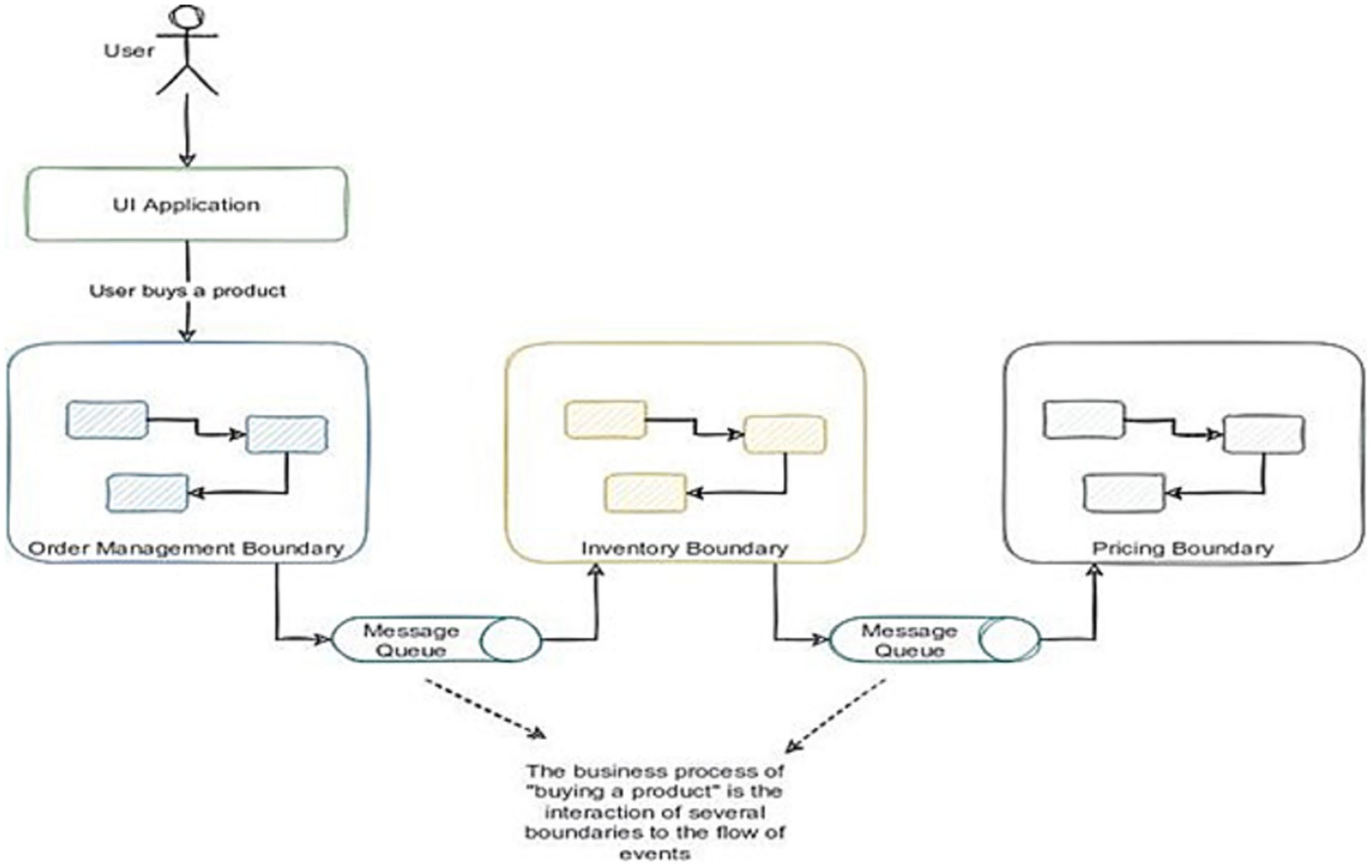
Communication of software services based on the event driven architecture pattern (Rocha, 2021).

Event-driven architecture (EDA) provides an architectural design capable of growing as architectures, such as services/microservices or even using Functions as a Service (FaaS). This framework enables organizations to effectively utilize the events generated at the technological level (refer to Figure 3).

### 4.6 Microkernel architecture

In a plugin-based architecture, characteristics are denoted and play a crucial role in delineating responsibilities. This ensures that the code remains organized and does not accumulate in processes that fail to meet the minimum requirements for sufficient quality.

For software to qualify as a plugin-based architecture, two main characteristics are required:

- To have a core that fully meets the conditions of being the main connector between the plugins and the main software, which will have the minimum vital for the system to exist even without plugins and produce value in the business logic as such, the project is divided into different tools that provide the value to the core to ensure that they can increase the capacity to perform different tasks.
- A plugin, this can be 0. This is owing to the system’s adaptability to withstand the minimum built by the project capacity that benefits the disconnection of such elements within the project, as well as adding the element.

## 5 Materials and methods

The process of constructing a software implementation involves defining clear system components and organizing development stages to ensure automation in the system’s business logic. ResNet50, a 50-layer convolutional neural network pre-trained on extensive image datasets, was employed for image recognition due to its robust feature extraction capabilities and proven effectiveness in handling large-scale visual data. Celery and Redis were utilized for efficient asynchronous task management, with Celery orchestrating task execution and Redis serving as a fast, in-memory data store to manage task queues and states. These technologies were selected after evaluating alternatives such as VGGNet and RabbitMQ, which displayed limitations in accuracy and scalability under similar conditions.

The resulting software artifact fulfills non-functional baseline requirements and automates essential processes. It ensures scalability and adaptability, allowing developers to continue building on the system without encountering mismatches in technology integration. Additionally, the software supports continuous integration (CI) and continuous deployment (CD) practices, ensuring consistent quality and readiness for end-customer environments. Security measures such as secret environment variables and user authentication were implemented to protect sensitive data.

This methodology provides a comprehensive approach to leveraging computer vision and MDA for automated code generation. The proposed framework facilitates a seamless transition from system design to implementation by addressing the identified gaps in software development.

Such a final software artifact should have the following:

- Auto-generated development code within the fundamental scopes integrated into the core business logic, utilizing methods such as ResNet50 for image recognition and Celery with Redis for task management. These choices were guided by their superior performance in managing large datasets and asynchronous tasks compared to alternatives such as VGGNet or RabbitMQ, which revealed limitations in scalability and flexibility for this application.
- Security involves keeping the environment variables secret for the use organization in question and providing the client with authentication/authorization to use the prototype.
- Scalability allows developers to build continuously without issues or discrepancies related to the technology being used.
- Continuous Integration (CI) in DevOps is a modern development practice that can be validated and verified. This allows us to achieve a standard of software quality (de Oliveira et al., 2023).
- DevOps Continuous Deployment (CD) is a practice focused on usability and the capability to be in environments ready for end customers (de Oliveira et al., 2023).
- Self-documentation capabilities allow for easier integration for any software developer or customer.
- Ability to grow to plugin level with the capability to integrate almost any programming language output through a respective template.
- Use of a version control system (VCS) for code maintenance and version management while taking advantage of the benefits it provides.

### 5.1 Scope and limitations

In relation to the services associated with the image interpretation process, the essential element to consider is image acquisition, for which local input devices will be employed, thereby enabling streaming to adjust the input image being captured, such as the local webcam and/or devices connected directly to the equipment executing the image input service appropriately. This is due to the fact that image quality significantly impacts the analysis. Similarly, in image processing and text recognition, the training process of the model can exhibit considerable uncertainty linked to the capacity of the equipment on which it is performed, including the GPU capability for neural network training and the RAM associated with image processing. Subsequently, the development encompasses several aspects beyond code generation, such as utilizing Docker for creating a container ready to be uploaded as a methodology for building the artifact that will house the application. The first iteration will implement the code generated within the software solution, leading to a repository on a cloud server focused on version control. However, these third-party services come with certain limitations, and if development exceeds basic coverage, adjustments must be made to accommodate these constraints. Additionally, thanks to the resources available at the open-source level, all tools used in the project offer free services that integrate seamlessly with the intended purpose of the process.

This project has no official or unofficial funding. Within each phase of development, implementation, and research, its execution is both scientific and technological, enabling the developer to utilize an Agile tool that is ready for the first sprint of SCRUM development in a startup.

### 5.2 Architecture

To fully develop the project, Clean Architecture can be utilized (Bass, 2023), which follows specific rules to ensure it is regarded as a viable solution with this design.

**Figure 4:**
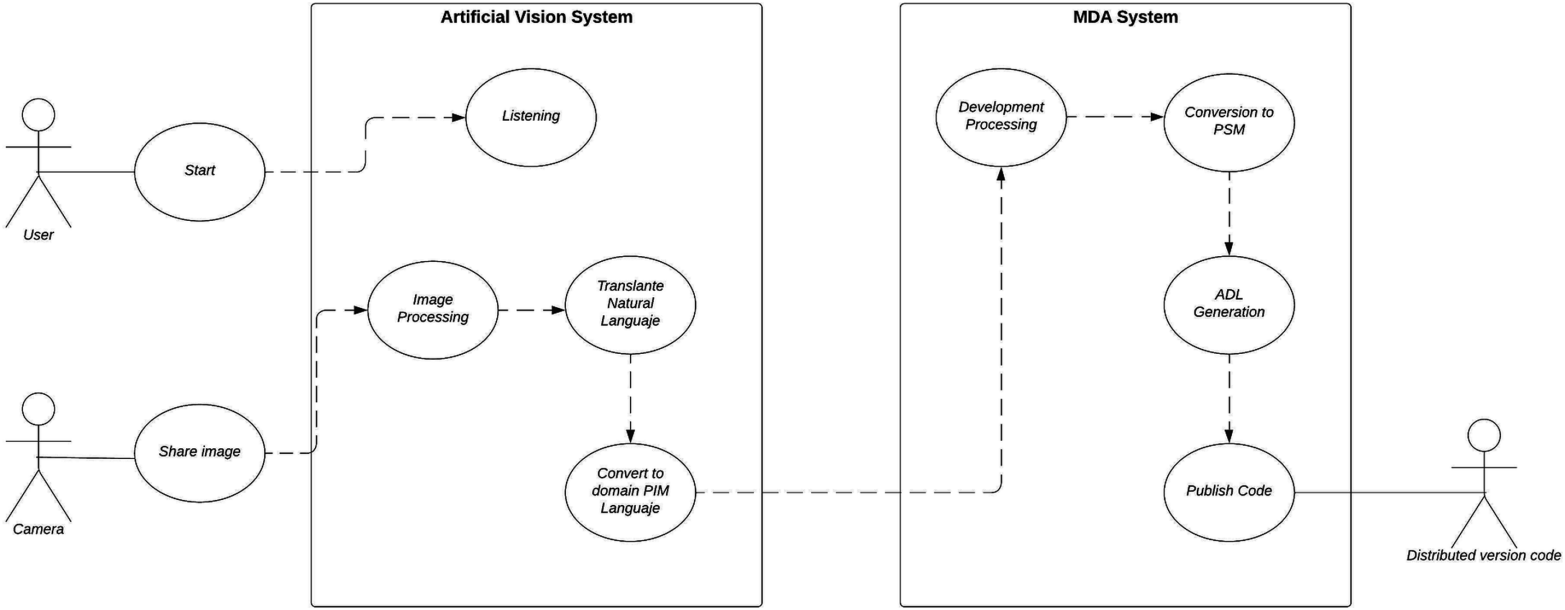
Implementation use case diagram. Own source.

The diagram below depicts the separation of the solution into two modules (Figure 4).

**Figure 5:**
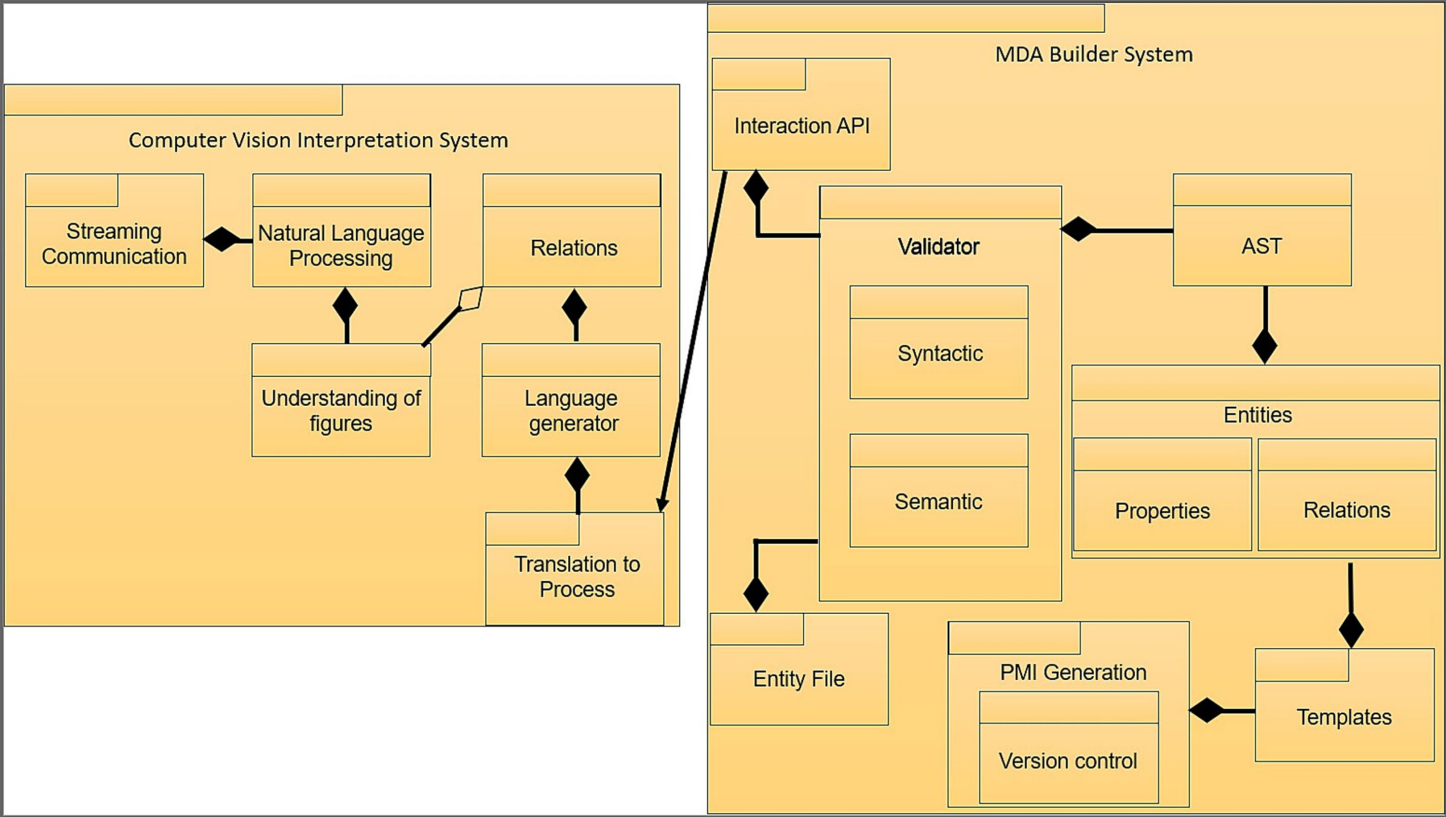
System diagram of the implementation. Own source.

This allows the creation of a system diagram, which serves as the primary design for the implementation (Figure 5).

### 5.3 Technology

In the process of selecting technology, it was essential to identify tools that effectively support asynchronous workflows, neural networks, machine learning, Model-Driven Architecture (MDA), and web development environments. Python has emerged as the optimal choice owing to its extensive ecosystem of libraries, including TensorFlow, Keras, and Celery, which facilitate rapid development and scalability. When compared to other programming languages such as Java or C++, Python’s simplicity and integration capabilities significantly diminish development overhead while sustaining robust performance. Furthermore, this decision capitalizes on Python’s strong support for domain-specific language (DSL) automation, a feature not as effectively supported by alternative ecosystems. Python was determined to be the most suitable technology due to its strong asynchronous capabilities and comprehensive library ecosystem, encompassing Keras, TensorFlow, and Celery, which enhance the development of machine learning models and automation processes. These characteristics present substantial advantages over alternatives such as Java or C++, which do not provide comparable simplicity and breadth of library support. Moreover, Python’s adaptability in managing domain-specific languages (DSLs) guarantees seamless integration and scalability, rendering it particularly appropriate for the project’s requirements.

GitHub was chosen as the version control platform due to its robust collaboration tools, extensive integration capabilities, and strong community support, making it ideal for maintaining and sharing project artifacts. Challenges such as managing multiple branches and ensuring synchronization between contributors during the integration process were addressed by implementing GitHub Actions for automated workflows. This allowed for seamless testing and deployment pipelines, significantly reducing manual intervention and errors. Notable successes included improved team collaboration and a streamlined process for code reviews and pull requests, enhancing code quality and project transparency. Heroku was selected for continuous deployment (CD) because of its simplicity, scalability, and seamless integration with GitHub, allowing rapid prototyping and deployment of software artifacts. However, challenges arose during deployment workflows, particularly in optimizing resource allocation and securely managing environment variables. These issues were mitigated by implementing automated monitoring tools and leveraging Heroku’s pipeline feature for better deployment tracking. Successes included faster iteration cycles and reduced downtime during deployments, significantly enhancing the development process. While other platforms such as GitLab and AWS could have been considered, GitHub and Heroku remained preferred for their user-friendly interfaces and alignment with the project’s agile development methodology. To address Heroku’s pricing model and resource limitations, deployment processes were optimized by limiting high-resource tasks during non-critical periods and leveraging scaling options to dynamically adjust resource use. Additionally, alternative platforms such as AWS Elastic Beanstalk were evaluated for specific scaling needs, ensuring the system remains cost-effective and adaptable for large-scale applications.

### 5.4 Development methodology

The project was built using an Agile methodology that integrates SCRUM and KANBAN (Schwaber, 2004) to execute it effectively, minimizing setbacks and ensuring all processes are applicable to the output system in question.

### 5.5 Temporal analysis

For the temporal analysis, the project will span 10 sprints, with 1 week allocated for each sprint.

## 6 Results and implementation

### 6.1 Infrastructure design

The construction of the implementation is based on the following infrastructure, utilizing a module for asynchronous tasks based on Celery and the Redis messaging broker.

**Figure 6:**
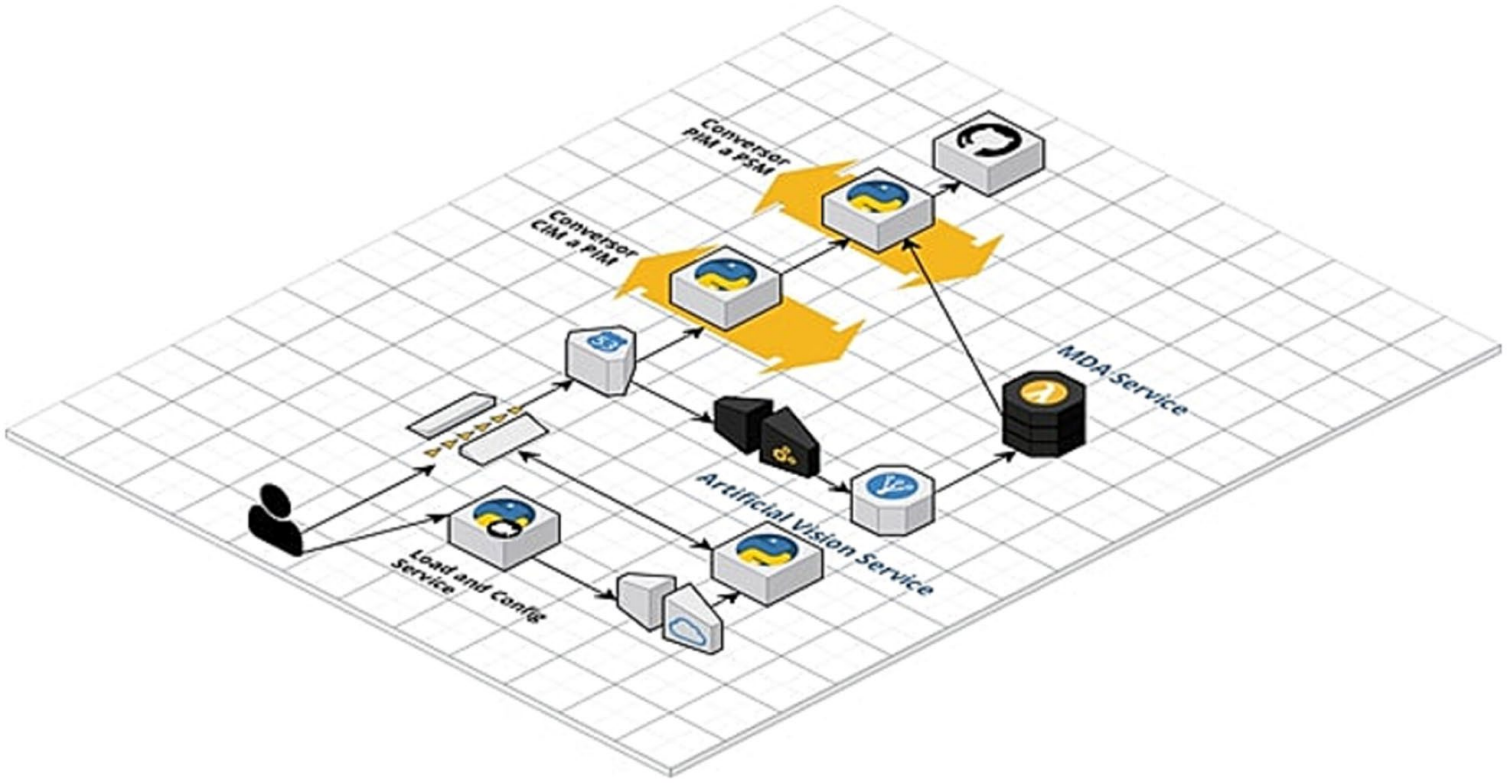
Architectural summary: development of the prototype. Own source.

It is determined by four stages, two of which correspond to the computational vision and processing domain and two to the MDA domain and source code generation (Figure 6).

### 6.2 Image recognition and processing

**Figure 7:**
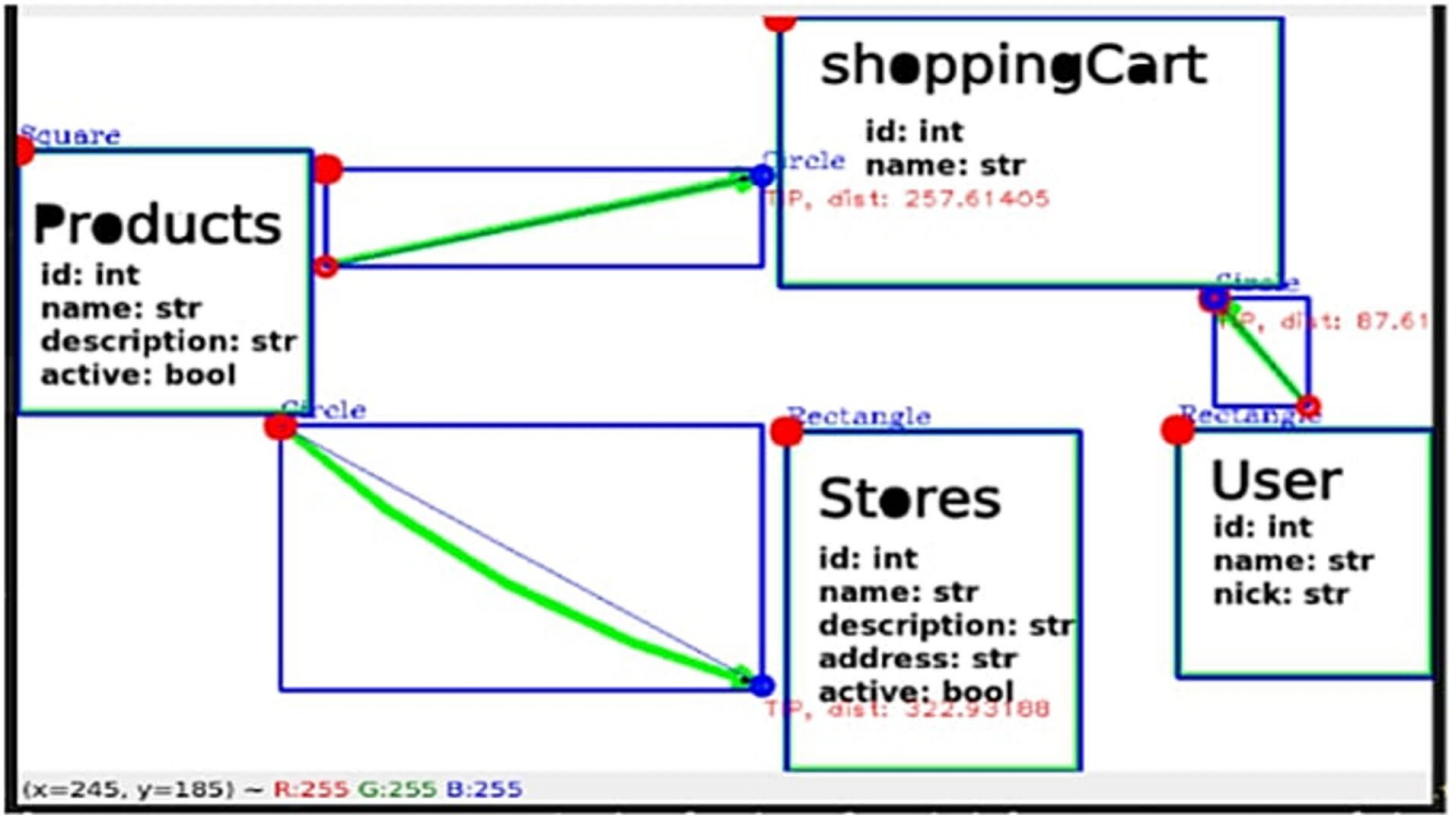
Image analyzed, with figures found (red), position of figures (blue dots and top left), defined outline of joints (green), endpoints (red dots), and tail of joints (blue dots at the end of the joints). Own source.

Initially, an image is essentially a matrix, as mentioned above. It corresponds to pixels, with each element indicating its color, which allows for information extraction. Processing the image is necessary to eliminate effects or elements that are not significant for interpretation. Various tools related to artificial or computer vision are utilized to define the important elements within the matrix, thus preserving the original images as original images:

1. Image noise removal: the image processing tool, Convolution, was employed, utilizing a bilateral filter that facilitates blurring while preserving edges and removing noise from the shadows of the image. We also apply techniques such as thresholding, which defines each pixel of an image based on a threshold value, enabling the highlighting of the most significant information within it. In this study, we used it adaptively to calculate the threshold in smaller regions, allowing us to define larger sets within the image. It is important to emphasize that this implementation shows improved effects on black-and-white images.
2. Defining and separating figures in the image: the Ramer–Douglas–Peucker algorithm was applied to the pre-processed image to approximate each contour detected in the figure, allowing differentiation between them with information such as the location or initial point from which it is defined.
3. Text analysis processes: in the processes related to analyzing text extracted from the pre-processed image, Optical Character Recognition (OCR) is employed to interpret text sourced from a computer. A Convolutional Neural Network is utilized to recognize manually drawn characters, the development process of which will be defined later.
4. Figure joining analysis: based on the aforementioned figure analysis and the respective positioning data previously obtained, it is possible to generate a bias of images from those containing text, resulting in a set of images that only define figures. By implementing the Ramer Douglas Peucker algorithm, the ends of the figures are identified as the areas with the highest concentration of points, from which the directionality of a drawn union can be established, along with the respective positioning of its start and end. Analyzing the centroids of the other figures (those with characters) allows us to identify those marked by both ends (Figure 7).

### 6.3 Neural network construction

**Figure 8:**
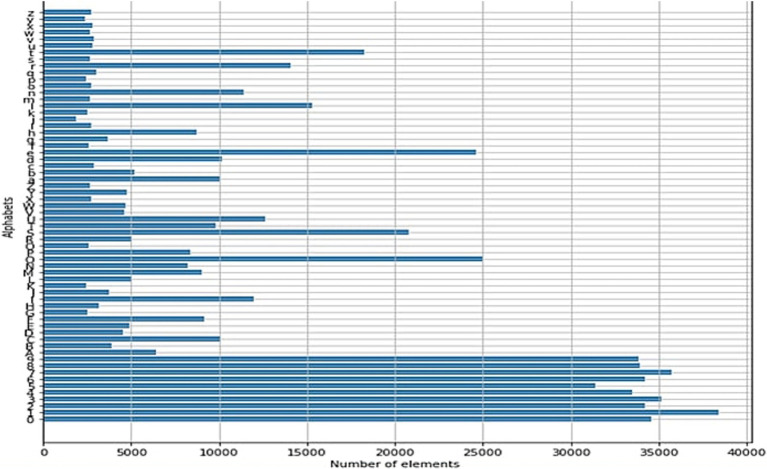
EMNIST training data count. Own source.

In building the neural network, the EMNIST dataset was used, which includes numbers as well as lowercase and uppercase letters, with a total of approximately 23,735,166 data points, as shown in (Figure 8).

The implementation of knowledge transfer is conducted using the pre-trained neural network model *ResNet50*. Therefore, adjusting the respective weights and re-entering the images from the EMNIST dataset is part of the limitations previously mentioned regarding the equipment used during the training process. This includes the necessary adjustment of the EMNIST dataset from 28 × 28 × 1 to 32 × 32 × 3 format required by ResNet50. Consequently, the training process, constrained by the teraflops limit, takes a long time, which contributes to one of the disadvantages explained later in the process. Thus, 20 epochs with 100,000 were utilized, allowing the generation of predictions based on the text derived from the entered image.

### 6.4 Data resulting from image interpretation

**Figure 9:**
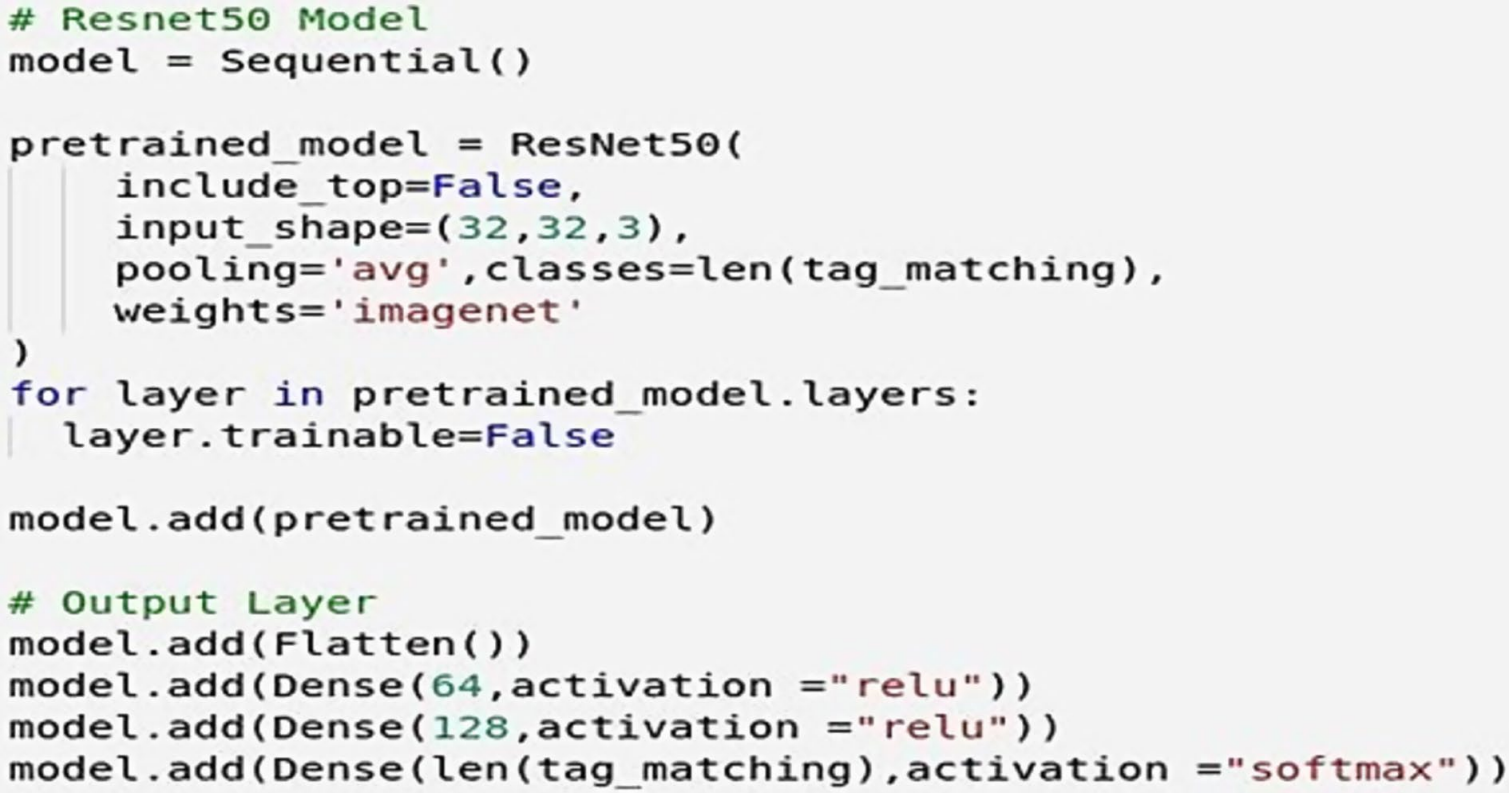
Sequential network with ResNet50 intermediate pre-training (knowledge transfer). Own source.

**Figure 10:**
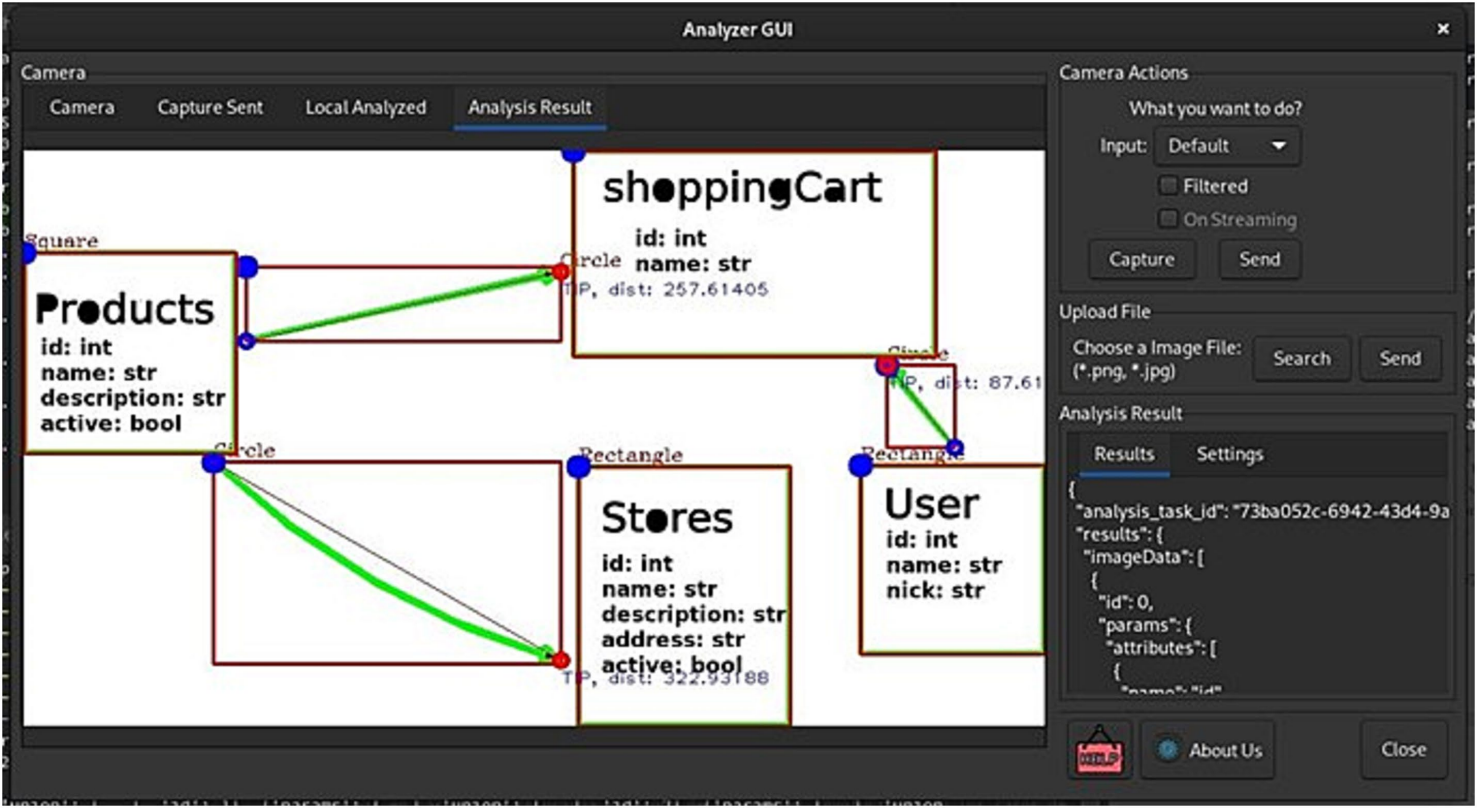
GUI for image input service and/or visualization of analysis results. Own source.

From the obtained data, a structure containing all the parameter information, as well as details about the model used for prediction, can be observed in the lower right section of the GUI (Figures 9, 10).

**Figure 11:**
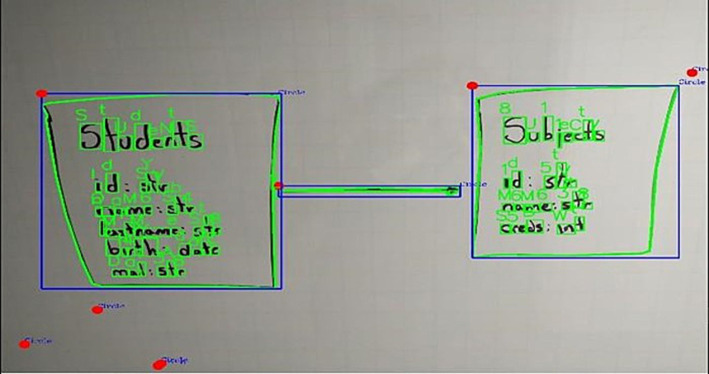
Diagram drawn and analyzed by the image processing service. Own source.

Similarly, when analyzing a drawn diagram, it can be observed (Figure 11).

### 6.5 Artifact generation and MDA

The domain-specific language was constructed to provide insight into the process of MDA entities and address the output to a point consistent with the output required by the system.

An example implementation of such a DSL that can be translated into the required process has the entities separated by type and size in memory if the data type requires it. The system’s default state, the null of the variable if allowed, and the interaction of each entity if connected by the business logic.

This interacts with one of the plugins that is prepared for generation and produces the resulting artifact: a repository on GitHub along with its corresponding deployment on Heroku.

### 6.6 Plugins

The tool allows several programming languages to be translated, and in the latest generation of this project, it will be utilized to facilitate interaction among all processes. It is built using plugins, setting each template aside. This is where technologies such as they enter an engine of views and extract results from the configurations provided in this process.

The interaction with the plugins works in a simple way from a plugins folder, where the files that allow the plugin/core connection are located.

This will allow the plugins to interact with their respective templates and generate the intended results based on the rules defined within the specific plugin configuration.

Each file contains the template configuration based on the Jinja view engine.

**Figure 12:**
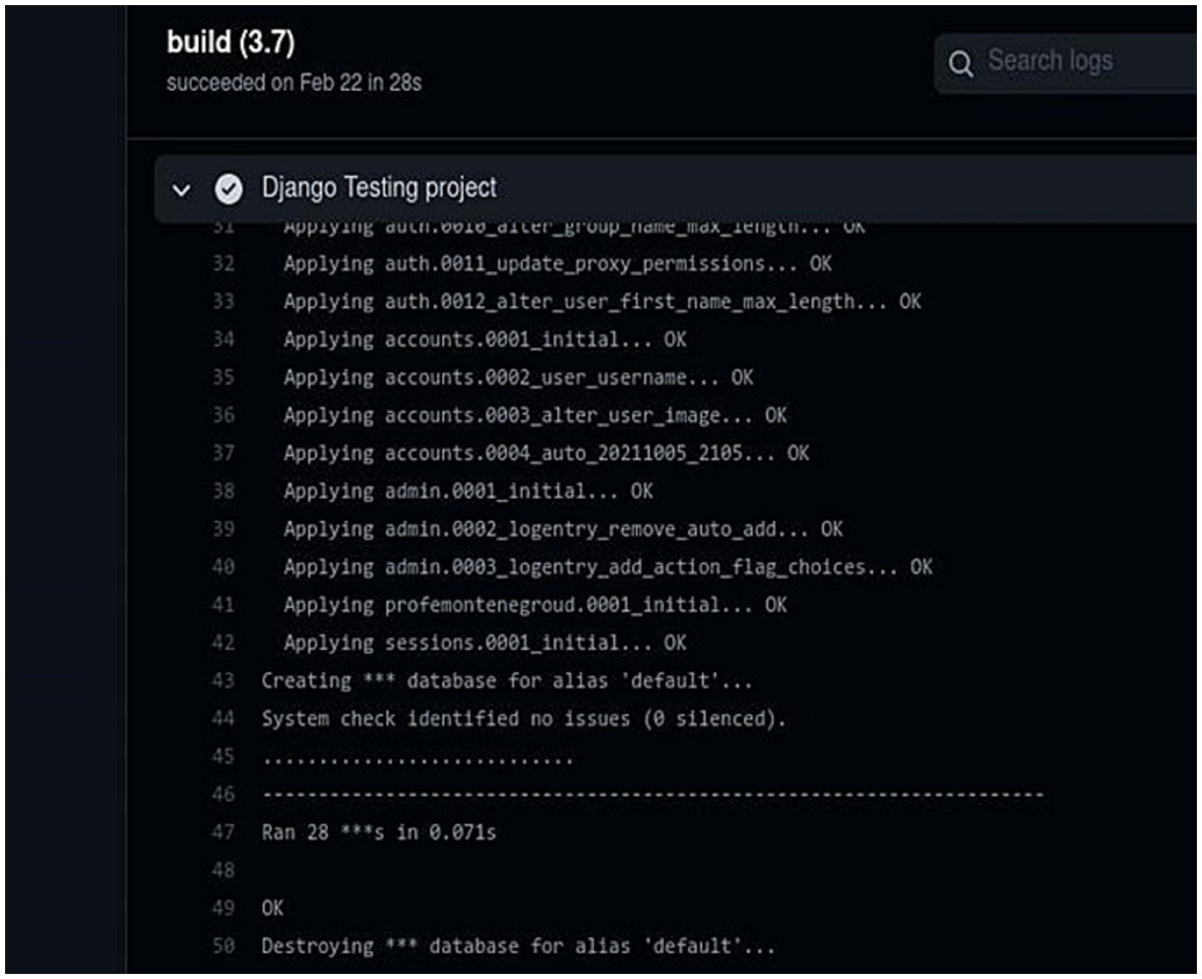
Quality verification of unitary testing code. Own source.

Submitting the DSL to the plugin generates a GitHub repository with the plugin’s source code and specifications (Figure 12).

Meanwhile, the events executed with the workflows of GitHub actions allow for the verification of the code and closure of the continuous integration (CI) process, in addition to the respective deployment in Heroku.

### 6.7 Disadvantages

The process of maintaining the deployment area secure involves various environment variables that contribute to a security solution. However, these variables can lead to problems even if they are auto-generated per project and encoded in base64. A certificate with OpenID is utilized for integration with Heroku, which requires two-step verification. For authorization, an OAuthV2 token is employed in both GitHub and Heroku, though it does not guarantee complete security in production. Therefore, it is essential to evaluate the extent of the process and the limitations that the generated tokens impose on the application’s security.

When improving results with the Convolutional Neural Network, the initial drawback is the capacity of the devices involved in the training process. This limitation arises when performing the initial training on a machine capable of reaching up to 768 GFLOPS and 16GB of RAM, which becomes overwhelmed by the volume of data from the EMNIST dataset (23,735,166 data). Reducing the dataset to 100,000 samples while utilizing the machine’s full capacity for an extended period leads to the reliance on *Google Colab*. Google Colab provides 13GB of free RAM and is equipped with an NVIDIA Tesla K80 GPU that delivers 1.87 Tflops, with a session limit of up to 12 h. Although this option significantly enhances training, it still faces time constraints. The training process is inherently limited and depends on the established amount of data, thus benefiting from greater physical resources.

**Figure 13:**
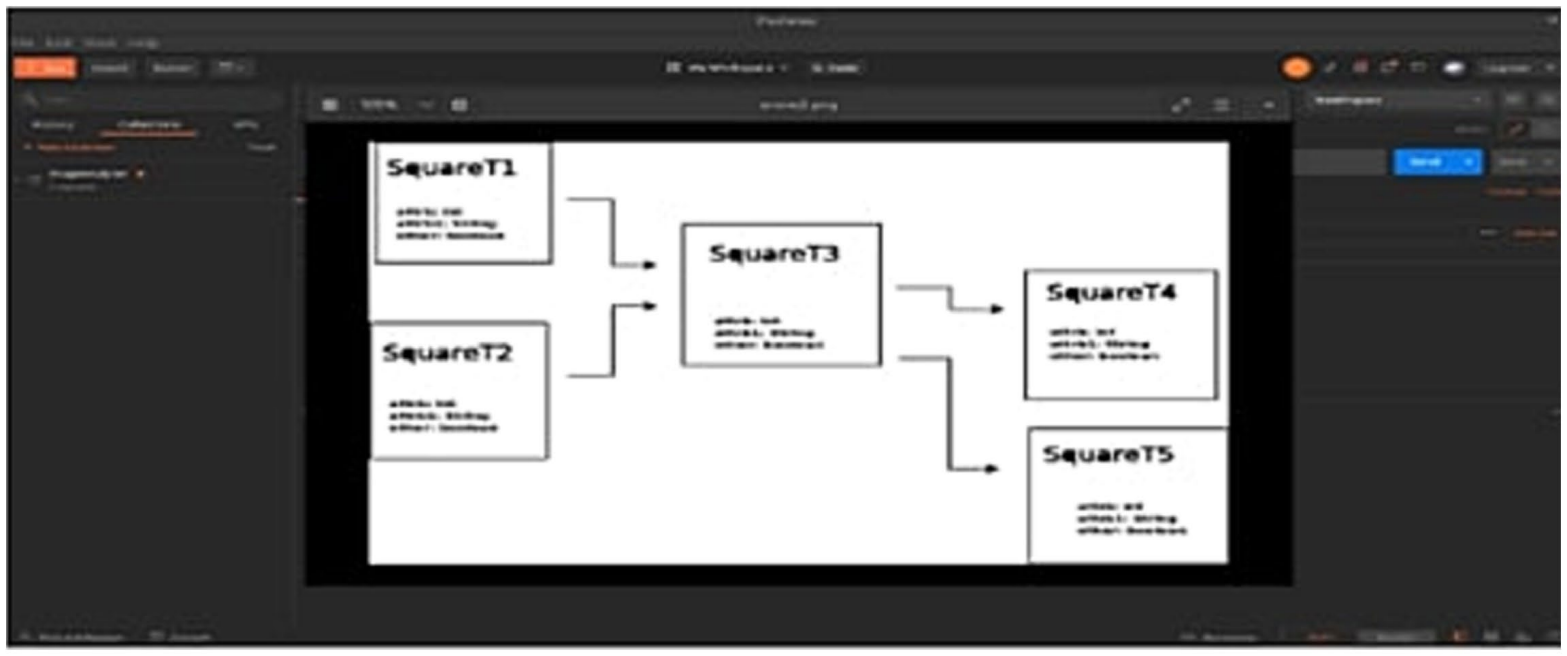
The original image is to be captured without the fisheye effect. Own source.

**Figure 14:**
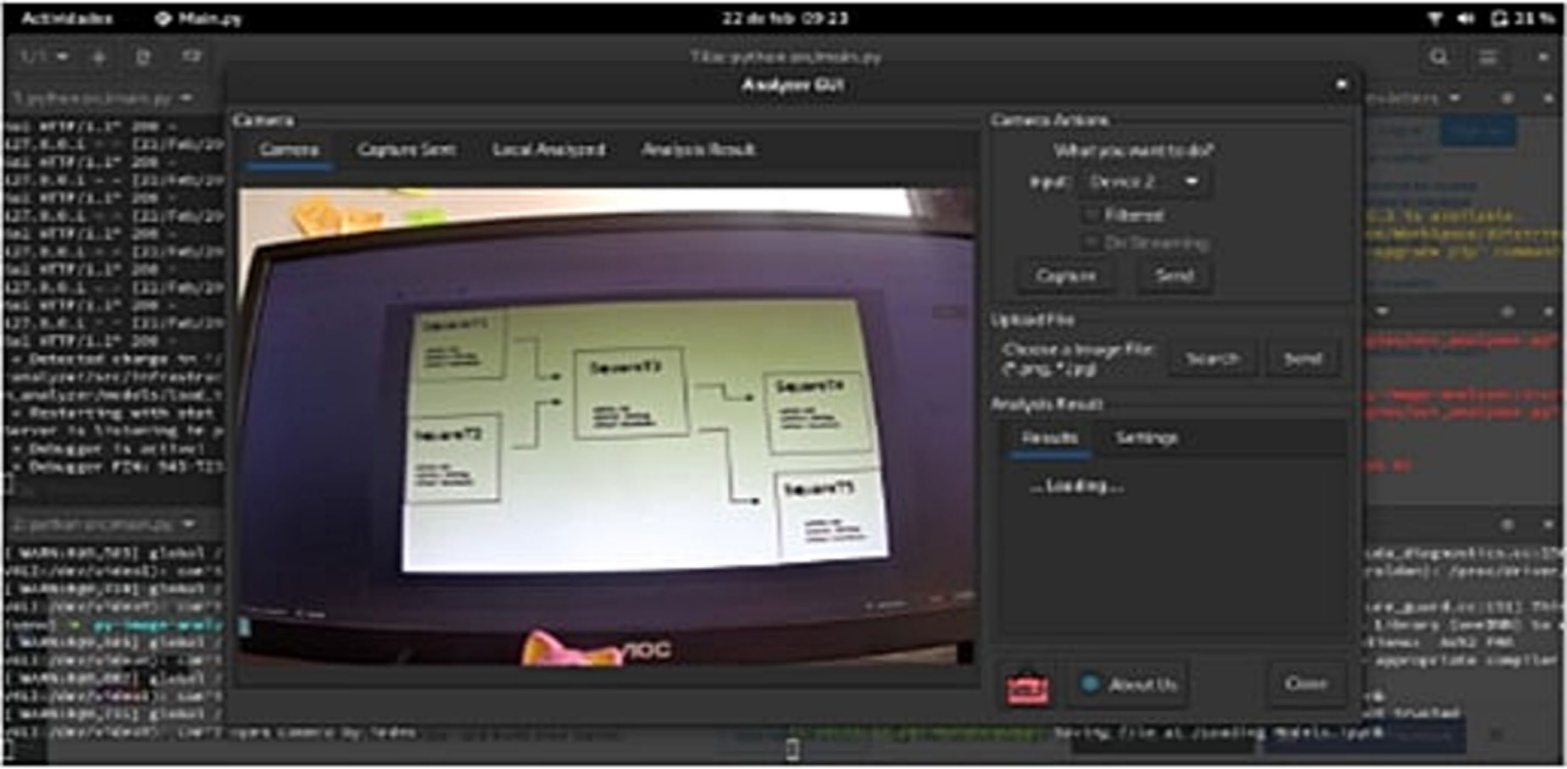
*Fisheye* effect on an input image. Own source.

**Figure 15:**
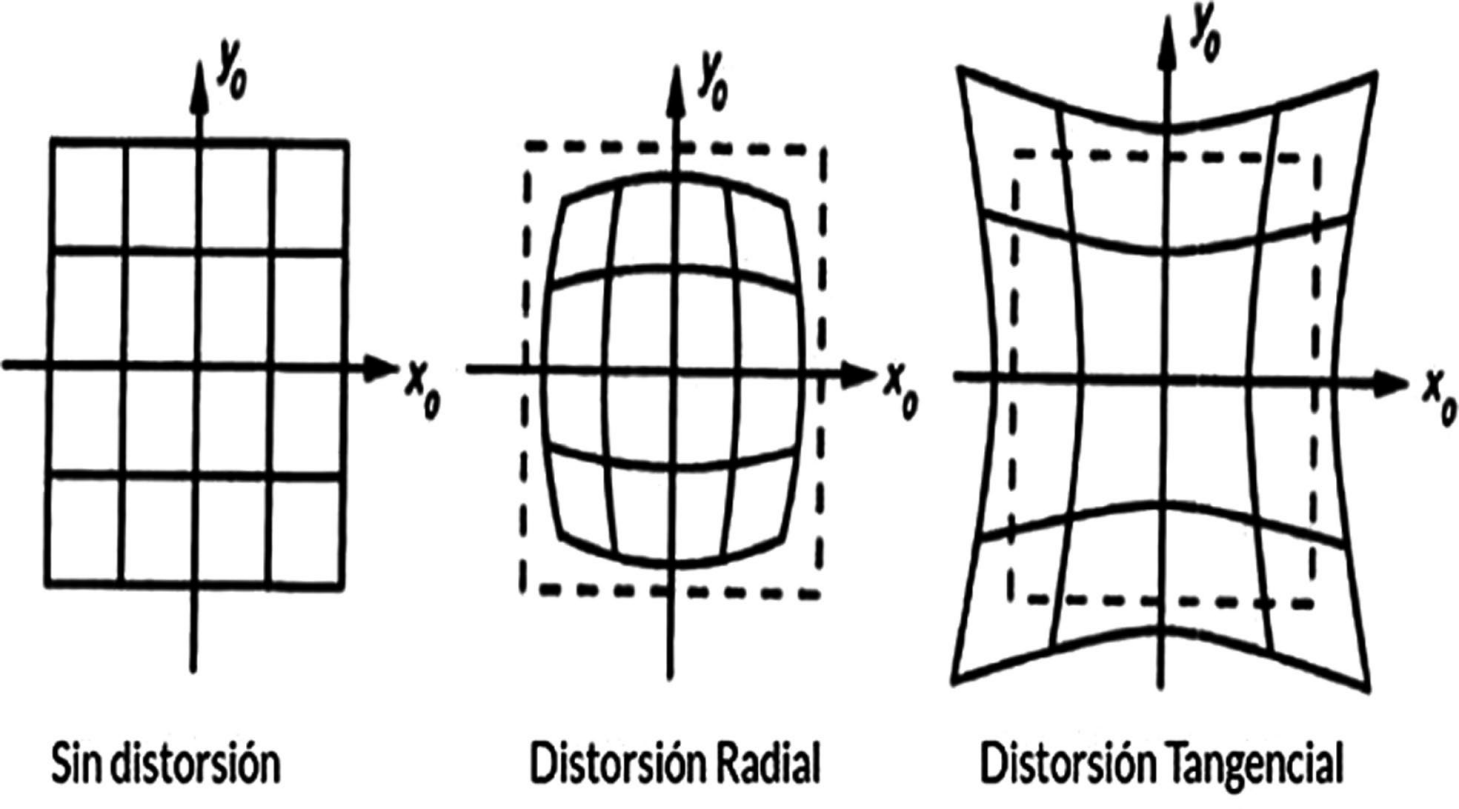
Effect of different distortions on the image. Own source.

In the process of capturing images with a connected camera (Figure 13), it becomes possible to observe a distortion process implemented by various cameras known as *fisheye*, which causes *radial distortion* to be readjusted during the camera calibration process (Figure 14). Thus, when readjusting using tangential distortion according to the axes in an undistorted image (Figure 15), there is a loss of data at the center of the image that, when linked to text processing, can significantly reduce the accuracy of predictions.

## 7 Discussion

The results of this study highlight the effectiveness of using computer vision and deep learning techniques for the automated generation of source code from architectural diagrams. The system effectively bridges the gap between the design and implementation phases by leveraging Model-Driven Architecture (MDA), providing a seamless mechanism to align business logic with functional requirements. However, the discussion requires broader contextualization to better position these findings within the larger landscape of code automation and AI-driven software engineering.

The MDA holds significant promise in transforming software development by automating processes that have traditionally relied on manual intervention. Its ability to translate high-level design artifacts into executable solutions is a game-changer, reducing human error and accelerating development timelines. Nevertheless, the challenges of implementation and the comparative advantages over alternative frameworks, such as Event-Driven Architecture (EDA) or Domain-Driven Design (DDD), require further exploration. For instance, while MDA excels at aligning abstract models with execution, its dependence on accurate model definitions can limit scalability in dynamic, rapidly evolving environments. Addressing these limitations through adaptive model interpretation and continuous learning could enhance its applicability.

In terms of future opportunities, the integration of advanced AI techniques such as reinforcement learning and generative adversarial networks (GANs) could open new frontiers for intelligent automation. These technologies can augment the system’s capabilities by enabling predictive adjustments to architectural changes, thereby improving adaptability. Moreover, incorporating real-time analytics into the development workflow could provide actionable insights, enhance decision-making, and foster more agile development practices.

The study emphasizes modular design, asynchronous task handling, and plugin-based extensibility, which provide a robust foundation for scalability. However, the implications of these architectural choices on industry adoption remain underexplored. For example, while using Celery and Redis enhances task efficiency, the scalability of these tools in large-scale, distributed environments warrants further investigation. Comparing this approach with cloud-native solutions such as AWS Lambda or Azure Functions could yield valuable insights into optimizing performance and cost-effectiveness.

The prototype achieved 85% accuracy in diagram interpretation and averaged a processing speed of 150 diagrams per hour, with error rates below 5%. This academic experiment evaluated the feasibility of integrating MDA with neural networks for automated code generation. Initial scalability tests demonstrated stable performance with up to 10,000 diagrams per session, showcasing the adaptability of the system’s modular architecture for future enterprise-scale applications. Challenges such as limited GPU capabilities were addressed by using cloud-based platforms and pre-processing techniques, ensuring robustness and providing insights into the optimization needs for broader use cases.

Future enhancements include enhancing parallel processing and refining the neural network architecture to manage computational constraints more effectively. These results underscore the potential of integrating MDA with AI techniques for automating software workflows, paving the way for practical and scalable implementations in various programming environments.

Finally, the discussion would benefit from a systematic examination of the future opportunities highlighted in the conclusions. These include the potential for integrating DevOps pipelines with AI-driven testing frameworks, which could further automate and enhance quality assurance processes. Additionally, exploring the ethical implications of automating code generation—such as ensuring transparency and accountability in generated artifacts—is an area that warrants attention as this technology matures.

## 8 Conclusion

This study generated the concepts of using MDA and microkernel architecture. The focus of AI is on computer vision, including issues related to Natural Language Processing (NLP). Third-party integrations applying DevOps, Clean Code, and Clean Architecture principles enable the implementation of a software solution that allows for the capture, processing, and translation of images, followed by their conversion to an intermediate and scalable meta-language implementation through various plugins. A version control system (VCS) facilitates the automatic packaging and deployment of a fully functional software artifact. All code quality conditions required for new changes made by the user to its entities can be managed appropriately without disrupting previous processes. Similarly, it is fundamental to consider the different processing constraints associated with various network training processes that can be enhanced through the implementation and/or training of networks on superior hardware, along with the limitations of machine processing where the execution of analysis and image processing occurs. This is evident in the different services involved in the final deployment of a completed project.

Meanwhile, during the progress of the project, its future implications and potential impact were analyzed. This yields certain results. Although the project meets the established objectives, its likelihood of realization in the future is remarkably high. The development, as observed in the industry, could change by basing its construction on design diagrams and models, which are fundamental to software development, recognizing the design stage not merely as one of the critical steps toward a software solution but as the most vital stage. This represents a breakthrough in terms of automation, translation, reading, and several other areas that could constitute a scientific paper on their own, as enunciated in this project. There is a significant opportunity to fulfill all the conditions outlined by the process.

## Data Availability

Requests to access datasets should be directed to apdazac@udistrital.edu.co.
